# Predictors of willingness to get a COVID-19 vaccine in the U.S

**DOI:** 10.1186/s12879-021-06023-9

**Published:** 2021-04-12

**Authors:** Bridget J. Kelly, Brian G. Southwell, Lauren A. McCormack, Carla M. Bann, Pia D. M. MacDonald, Alicia M. Frasier, Christine A. Bevc, Noel T. Brewer, Linda B. Squiers

**Affiliations:** 1 RTI International, Research Triangle Park, 701 13th St. NW, Ste. 750, Washington, DC 20005 USA; 2 Gillings School of Global Public Health, University of North Carolina, Chapel Hill, North Carolina USA

**Keywords:** COVID-19 vaccine, SARS-CoV-2 vaccine, Vaccine hesitancy, Public health communication

## Abstract

**Background:**

As COVID-19 vaccine distribution efforts continue, public health workers can strategize about vaccine promotion in an effort to increase willingness among those who may be hesitant.

**Methods:**

In April 2020, we surveyed a national probability sample of 2279 U.S. adults using an online panel recruited through address-based sampling. Households received a computer and internet access if needed to participate in the panel. Participants were invited via e-mail and answered online survey questions about their willingness to get a novel coronavirus vaccine when one became available. The survey was completed in English and Spanish. We report weighted percentages.

**Results:**

Most respondents were willing to get the vaccine for themselves (75%) or their children (73%). Notably, Black respondents were less willing than White respondents (47% vs. 79%, *p* < 0.001), while Hispanic respondents were more willing than White respondents (80% vs. 75%, *p* < 0.003). Females were less likely than males (72% vs. 79%, *p* < 0.001). Those without insurance were less willing than the insured (47% vs. 78%, *p* < 0.001). Willingness to vaccinate was higher for those age 65 and older than for some younger age groups (85% for those 65 and older vs. 75% for those 50–64, *p* < 0.017; 72% for those 35–49, *p* < 0.002; 70% for those 25–34, *p* = NS and 75% for ages 18–24, *p* = NS), but other groups at increased risk because of underlying medical conditions or morbid obesity were not more willing to get vaccinated than their lower risk counterparts.

**Conclusions:**

Most Americans were willing to get a COVID-19 vaccine, but several vulnerable populations reported low willingness. Public health efforts should address these gaps as national implementation efforts continue.

## Introduction

As nations across the globe continue to distribute COVID-19 vaccinations to eligible individuals, some health officials argue that the time is fast approaching when issues with logistics and short supply will be replaced by the need to reach those who are more reluctant to vaccinate [1, 2]. A global study found that 72% of people would take a Covid-19 vaccine if proven safe and effective, but willingness varied widely between nations [3]. Studies in Australia and Indonesia have found that willingness varies widely depending on the vaccine’s effectiveness and that safety is a concern [4, 5].

Given the unique features of this particular virus—its highly contagious nature, high severity for certain groups, and the significant impact it has had on people’s daily lives through social distancing and business closures—we sought to better understand the American public’s willingness to get a COVID-19 vaccine. We also sought to characterize willingness among groups most at risk for severe COVID-19 (e.g., people age 65 or older, those with specific underlying medical conditions, or with a body mass index (BMI) > 40; and Black and Hispanic Americans) [6]. We examined these questions in a national survey of U.S. adults.

## Methods

### Participants and procedures

We surveyed U.S. households in April 2020 using a pre-recruited, address-based web panel consisting of 55,000 adults. The panel is based on probability sampling covering both the online and offline populations in the United States. Households received a computer and internet access if needed to participate in the panel. The resulting panel includes representation from listed and unlisted telephone numbers, telephone and non-telephone households, mobile phone–only households, and households with and without internet access. A random sample of panel members yielded 2279 survey respondents, for a stage completion rate of approximately 34% [7]. Participants were invited via e-mail. The survey was completed online, in English for most participants; 227 respondents completed it in Spanish. RTI International’s Institutional Review Board designated the study protocol as exempt from human subjects approval. All methods were carried out in accordance with relevant guidelines and regulations. Informed consent was obtained from all participants.

### Measures

The survey assessed willingness to receive a vaccine when one becomes available, beliefs about the virus, and demographic characteristics (Table 1).

**Table 1:** Survey measures related to novel coronavirus and potential vaccination

Type of variable	Construct	Question	Response options (and recoding)
Outcomes	Willingness to receive the vaccine	When a vaccine for the Coronavirus becomes available, I will get it.	Strongly disagree; disagree; agree; strongly agree (recoded as willing or not willing)
	Willingness to vaccinate the child	When a vaccine for Coronavirus becomes available, I will have my child get it.	Strongly disagree; disagree; agree; strongly agree (recoded as dichotomous)
Predictors	Underlying medical conditions	Who in your household has any of the following: -Chronic kidney disease (undergoing dialysis) or liver disease (e.g., cirrhosis, chronic hepatitis)? -a compromised immune system (immunosuppression)? -diabetes? -a serious heart condition? -chronic lung disease? -moderate to severe asthma?	Myself; someone in my household other than me; no one (for this measure, we used only respondents’ answers about themselves)
	Worry	I am worried about getting the Coronavirus	Strongly agree; agree; disagree; strongly disagree
	Perceived threat	What level of threat do you think the Coronavirus poses to each of the following? you or your family (Other objects of threat were included on the survey but will be reported elsewhere.)	Very high threat; high threat; moderate threat; low threat; very low threat; don’t know (Recoded into a four-level item: low/very low; moderate; high/very high; don’t know)
	Past flu vaccination behavior	When was the last time you were vaccinated for the flu?	Less than 1 year ago; 1–2 years ago; 3 or more years ago; or I have never been vaccinated for the flu

### Analyses

We report unweighted frequencies and weighted percentages and odds ratios. A post-stratification process was used to adjust for survey nonresponse and any noncoverage, undersampling, or oversampling resulting from the study-specific sample design. The panel provider used data from the U.S. Census Bureau (https://www.census.gov/programs-surveys/cps.html) to weight all respondents to benchmarks for gender, age, race/ethnicity, education level, Census region, household income, metropolitan area, and homeownership status.

Weighted multivariable logistic regression examined predictors of willingness to vaccinate oneself and one’s child (dichotomized as willing vs. not willing). Predictor variables were gender, education, race/ethnicity, Hispanic origin, health insurance, income, previous seasonal influenza vaccination, COVID-19 severe disease risk factors (age 65+, BMI > 40, chronic health conditions), perceived threat of the virus, and worry. Critical alpha for statistical significance was set at .05.

## Results

Twenty-two percent of participants were age 65 or older (see Table 2). Seventeen percent had a BMI over 40, and 26% had one or more underlying medical conditions that could put them at higher risk of severe illness from the virus. Fifty-four percent had received the seasonal influenza vaccine in the last year, while 18% reported that they had never received the seasonal influenza vaccine. Twenty-seven percent of respondents had children.

**Table 2:** Participant characteristics (*n* = 2247)

Characteristics	Number of Individuals	Weighted %
**Demographics**		
Gender		
Male	1158	48
Female	1089	52
Education		
< High school	190	11
High school	578	28
Some college	611	28
Bachelor’s degree or higher	868	34
Race/ethnicity		
White	1876	78
Black	183	12
Other	188	10
Hispanic origin		
Yes	441	16
No	1806	84
Income, annual		
< $25,000	253	13
$25,000–$49,999	408	18
$50,000–$99,999	729	31
$100,000–$149,999	394	16
≥ $150,000	463	21
Employed		
Yes	1489	65
No	758	35
Health insurance		
Employer/union	1044	44
Medicare/Department of Veterans Affairs	455	20
Medicaid	132	7
Other	144	6
None	130	6
Don’t know/refused	342	16
Has children age 0–18		
Yes	603	27
No	1644	73
Received seasonal influenza vaccine in past year		
Yes	1245	54
No	994	46
Unknown	8	0
**Severe COVID-19 risk factors**		
Age, years		
18–24	186	10
25–34	351	17
35–49	531	24
50–64	668	26
65+	511	22
Had a chronic medical condition		
Yes	584	26
No	1582	71
Unknown	81	4
Severe obesity (BMI > 40)		
Yes	395	17
No	1740	78
Unknown	112	5
**Risk appraisals**		
Worried about getting the novel coronavirus		
Strongly agree	453	20
Agree	1070	47
Disagree	537	24
Strongly disagree	178	8
Unknown	9	1
Perceived threat from novel coronavirus		
High/very high	512	24
Moderate	911	40
Low/very low	738	32
Don’t know	82	4
Refused	4	0

*Note*: *BMI* body mass index, *REF* reference group

Overall, 75% were willing to get a COVID-19 vaccine when it becomes available (37% strongly agreed and 38% agreed). Willingness for their children was similar, at 73% (32% strongly agreed and 41% agreed). Asked about the level of threat from the virus posed to self and family, 8% answered “very high,” 15% answered “high,” 40% said “moderate,” and 33% said “low or very low.” 4% did not know. Sixty-eight percent reported being worried about getting the novel coronavirus.

### Associations with willingness

Black respondents were less willing to get the vaccine than White respondents (53% vs. 79%, OR = 0.34, 95% CI = 0.22–0.54, Table 3). Willingness to get vaccinated was also lower among respondents with the lowest annual incomes (<$50,000 OR = 0.66, 95% CI = 0.44–1.01; $50,000–$99,999 OR = 0.65, 95% CI = 0.44–0.97; ≥$150,000 = REF) and those without health insurance (OR = 0.44, 95% CI = 0.27–0.72). Willingness to get the vaccine for oneself was higher for male than female adults (79% vs. 72%, OR = 1.56, 95% CI = 1.20–2.03) and lower for those with less education.

**Table 3:** Results of logistic regressions for willingness to get vaccine for self and child

Variable	Willingness for Self			Willingness for Child		
	*n* (Wtd %)	Adjusted OR (95% CI)	*P*	*n* (Wtd %)	Adjusted OR (95% CI)	*p*
Gender						
Male	932 (78.9)	1.56 (1.20, 2.03)	0.001	645 (73.5)	1.32 (1.01, 1.75)	0.045
Female	805 (72.3)	REF		557 (68.1)	REF	
Age, years						
18–24	144 (75.3)	0.89 (0.52, 1.50)	0.655	98 (73.6)	1.00 (0.56, 1.78)	0.991
25–34	253 (69.7)	0.65 (0.41, 1.02)	0.063	165 (62.7)	0.57 (0.36, 0.90)	0.017
35–49	387 (71.6)	0.52 (0.34, 0.79)	0.002	317 (69.2)	0.58 (0.38, 0.88)	0.010
50–64	510 (74.8)	0.62 (0.42, 0.92)	0.017	348 (69.1)	0.63 (0.42, 0.94)	0.024
65+	443 (84.9)	REF		274 (80.4)	REF	
Education						
High school or less	542 (68.7)	0.55 (0.38, 0.79)	0.001	382 (63.7)	0.54 (0.37, 0.79)	0.001
Some college	457 (73.5)	0.65 (0.47, 0.91)	0.011	326 (70.5)	0.77 (0.55, 1.09)	0.146
Bachelor’s degree or higher	738 (84.8)	REF		494 (79.3)	REF	
Race/ethnicity						
White	1498 (79.0)	REF		1024 (74.4)	REF	
Black	100 (53.3)	0.34 (0.22, 0.54)	<.001	75 (48.1)	0.34 (0.22, 0.52)	<.001
Other	139 (74.3)	0.65 (0.40, 1.05)	0.080	103 (72.3)	0.76 (0.45, 1.28)	0.296
Hispanic origin						
Yes	352 (80.2)	1.72 (1.20, 2.46)	0.003	301 (79.4)	1.89 (1.31, 2.72)	<.001
No	1385 (74.5)	REF		901 (68.6)	REF	
Income						
< $50,000	466 (67.8)	0.66 (0.44, 1.01)	0.057	321 (62.0)	0.55 (0.35, 0.87)	0.011
$50,000–$99,999	557 (74.2)	0.65 (0.44, 0.97)	0.033	380 (69.6)	0.66 (0.43, 1.00)	0.048
$100,000–$149,999	325 (81.4)	0.97 (0.61, 1.55)	0.912	232 (78.4)	1.02 (0.63, 1.65)	0.940
≥ $150,000	389 (84.1)	REF		269 (80.1)	REF	
Health insurance						
Insured	1402 (77.8)	REF		947 (71.9)	REF	
Not insured	69 (46.7)	0.44 (0.27, 0.72)	0.001	50 (49.0)	0.64 (0.37, 1.12)	0.118
Don’t know/refused	266 (75.5)	1.15 (0.78, 1.69)	0.490	205 (73.3)	1.46 (1.00, 2.15)	0.053
Received flu vaccine in past year						
Yes	1132 (89.5)	4.70 (3.55, 6.23)	<.001	773 (83.3)	3.13 (2.37, 4.14)	<.001
No	601 (58.9)	REF		426 (56.0)	REF	
Had a chronic medical condition						
Yes	473 (79.4)	0.85 (0.62, 1.15)	0.291	315 (73.3)	N/A	
No	1199 (73.7)	REF		845 (69.7)		
Severe obesity (BMI > 40)						
Yes	311 (77.3)	1.09 (0.79, 1.50)	0.614	210 (70.8)	N/A	
No	1338 (75.0)	REF		932 (70.7)		
Worried about getting the coronavirus						
Strongly agree	398 (86.2)	5.37 (3.10, 9.29)	<.001	290 (82.3)	5.70 (3.21, 10.13)	<.001
Agree	891 (81.9)	3.96 (2.51, 6.25)	<.001	606 (77.1)	4.56 (2.76, 7.52)	<.001
Disagree	365 (65.7)	1.82 (1.14, 2.88)	0.011	254 (60.4)	2.20 (1.35, 3.57)	0.002
Strongly disagree	81 (43.8)	REF		50 (38.9)	REF	
Perceived threat from the coronavirus						
High/very high	437 (82.3)	1.46 (0.99, 2.16)	0.054	316 (80.4)	1.82 (1.21, 2.72)	0.004
Moderate	742 (80.2)	1.42 (1.04, 1.94)	0.026	504 (73.4)	1.21 (0.88, 1.66)	0.247
Low/very low	520 (68.7)	REF		349 (62.3)	REF	
Don’t know/Refused	36 (37.4)	0.33 (0.17, 0.63)	0.001	30 (49.1)	0.80 (0.41, 1.56)	0.514

*Note*. Analysis of vaccination willingness for self included 1737 respondents. Analysis of vaccination willingness for child included 1202 respondents. *BMI* body mass index, *OR* odds ratio, *REF* reference group, *Wtd* weighted

As for others in high-risk groups, willingness was higher for those age 65 or older, and Hispanics compared to other respondents (Table 2). However, those with underlying medical conditions and BMI > 40 were not more willing to get vaccinated than those without these risk factors.

Willingness to get vaccinated was higher among those who were worried about getting the novel coronavirus (strongly agree 86%, OR = 5.37, 95% CI = 3.10–9.29; agree 82%, OR = 3.96, 95% CI = 2.51–6.25; disagree 66%, OR = 1.82, 95% CI = 1.14–2.88) as compared to those least worried (strongly disagree = 44%). Willingness to get vaccinated was higher among those with moderate perceived threat (80%; OR = 1.42, 95% CI = 1.04–1.94) and lower among those who answered “don’t know” about the threat to themselves or family (37%; OR = 0.33, 95% CI = 0.17–0.63) than among those with the lowest level of threat (69%). High perceived threat was not statistically significant.

Willingness to get vaccinated was higher among those who received a seasonal influenza vaccine in the past year than those who had not (90% vs. 59%, OR = 4.70, 95% CI = 3.55–6.23). Willingness to vaccinate children showed a similar pattern of results (Table 3).

## Discussion

In a large nationally representative survey conducted in April 2020, the willingness of U.S. adults to receive a novel coronavirus vaccine was high. Most U.S. adults were willing to get the vaccine for themselves and for their children, which is encouraging in a climate in which vaccine hesitancy around childhood immunizations has received global attention. Those most willing to get a COVID-19 vaccine were over age 65, had a bachelor’s degree or higher, were worried about the novel coronavirus, and had received an influenza vaccine in the previous year.

Our estimate of the percentage of people who indicated that they were not willing to get vaccinated is similar to other polls during the same time period (e.g., [8]), but our estimate of those willing to vaccinate was somewhat higher. One explanation is that our survey forced respondents to report willingness or unwillingness to get the vaccine because the item did not include “don’t know” or “not sure” response options. Thus, our findings suggest that those who report they are unsure in some polls may be willing to do so when pressed to decide. More research is needed to determine the conditions under which that willingness will translate into action when the time comes to vaccinate.

Studies during previous infectious disease outbreaks suggest somewhat lower willingness to get a new vaccine during a pandemic of a novel pathogen. During the 2009 influenza A (H1N1) pandemic, 64% of adults in two North Carolina counties intended to receive a vaccine when available [9]. In that study, seasonal influenza vaccination was associated with intentions to get the H1N1 vaccine, but other factors (H1N1 vaccine knowledge, age, gender, race/ethnicity, work status, and having children under age 18 living at home) were not. During the 2016 Zika virus pandemic, 56% of respondents in a national study were willing to get the hypothetical vaccine [10]. The higher willingness to obtain a COVID-19 vaccine than those for these two previous disease outbreaks may reflect higher media coverage and higher perceived threat of COVID-19.

Black respondents were markedly less willing than White respondents to get a COVID-19 vaccine, a finding that is consistent with adult vaccination trends of, for example, lower seasonal influence vaccine coverage in the same group [11]. Given that Black people have experienced disproportionately high rates of hospitalization and death from COVID-19 [12], the disparity in COVID-19 vaccine willingness raises the possibility that vaccination could amplify existing disparities. Respondents of Hispanic origin expressed higher willingness than their non-Hispanic counterparts to get the vaccine. Although adult vaccine coverage has generally been lower for Hispanics than non-Hispanic Whites in recent years [13], their greater willingness to get a COVID-19 vaccine may reflect the virus’s disproportionate impact on Hispanic communities (e.g., [14]). It will be imperative to ensure equitable access to the vaccine even as efforts address vaccine hesitancy among diverse communities. The upcoming report by the National Academies of Sciences, Engineering, and Medicine on this question will provide critical guidance for the nation on addressing disparities in vaccine distribution and uptake.

People with lower socioeconomic status were generally less willing to get vaccinated. Lower income and lower education respondents had less willingness to get the vaccine. Those without insurance were nearly 30% less willing to get the vaccine than insured respondents. Interventions aimed at reducing financial barriers to vaccination and increasing vaccine availability are among the most successful [15]. Equity-oriented policy initiatives focused on cost and access barriers will need to be coupled with communication efforts to make sure those who need them are aware of the options available to them.

### Implications for health communication

Our findings have implications for public health communication interventions.

Perceived and actual risk showed different patterns of association in our study. Individuals with higher risk appraisals—*perceived* threat and greater worry—were more willing to get vaccinated, a finding that is consistent with previous research [16]. However, willingness to get a vaccine was not always associated with being at *objectively* high risk for severe COVID-19. Individuals over age 65 were more willing than some younger age groups; however, individuals with underlying medical conditions and those with BMI > 40 were not more willing. Healthcare providers treating individuals with these conditions may have an opportunity to help them better understand their risk.

The public has been inundated with information about COVID-19. The question is no longer whether to communicate, but how to effectively provide accurate information to meet the vaccination needs of U.S. adults. The Extended Parallel Process Model [17] suggests that health messages are more likely to result in health behavior when they are threatening and lead people to believe the recommended behavior is effective at reducing the threat. Messages that emphasize both the significant threat of this virus to people most at risk and the effectiveness of a recommended vaccine (once that is known) could help persuade those who are on the fence about vaccination [18]. That message strategy paired with an audience segmentation approach that addresses those less willing or unsure about vaccination (e.g., those with lower education, those of Black race, and those without insurance) would be a good starting point for health communication campaigns.

### Strengths and limitations

Our study relied on a representative national sample of U.S. adults to quickly gather data on a pressing public health problem. We recognize that we were asking about a hypothetical behavior. Nonetheless, understanding how perceptions are changing can provide helpful information for public health planning and communication. Prior research has shown that behavioral willingness is highly correlated with actual behavior and may even predict behavior independent of intentions [19].

## Conclusions

The findings from this study provide direction about areas to focus public health planning and communication efforts as vaccine efforts continue.

Future research should focus on reasons for some of the differences we found as well as explore how communication about the virus and vaccination has changed perceptions since this survey was conducted in April 2020. These results offer a snapshot of public perceptions early in U.S. COVID-19 pandemic experience. As public perceptions change over time, we can expect communication to continue to play a critical role in generating confidence in the programs and policies that allow nations to achieve high vaccine uptake [15]. Social processes including provider recommendations, social norms, and social media sharing also likely play an important role in vaccine uptake. Our study shows great willingness among Americans to vaccinate and yet we also can see possibilities for notable differences between some groups that warrant efforts to mitigate health inequity.

## Data Availability

The dataset generated during the current study is not publicly available due to the fact that authors are still analyzing data on other survey topics and producing additional reports.
