# Correction to: Predictors of willingness to get a COVID-19 vaccine in the U.S

**DOI:** 10.1186/s12879-021-06085-9

**Published:** 2021-04-26

**Authors:** Bridget J. Kelly, Brian G. Southwell, Lauren A. McCormack, Carla M. Bann, Pia D. M. MacDonald, Alicia M. Frasier, Christine A. Bevc, Noel T. Brewer, Linda B. Squiers

**Affiliations:** 1 RTI International, Research Triangle Park, 701 13th St. NW, Ste. 750, Washington, DC, 20005 USA; 2 Gillings School of Global Public Health, University of North Carolina, Chapel Hill, North Carolina USA

**Correction to: BMC Infect Dis 21, 338 (2021)**

**https://doi.org/10.1186/s12879-021-06023-9**

In the original publication of this article [1] there was 1 typographical error in the abstract. The incorrect and correct information is listed below. The original article has been updated.

**Incorrect**

- Most respondents were willing to get the vaccine for themselves (75%) or their children (73%). Notably, Black respondents were less willing than White respondents (47% vs. 79%, *p* < 0.001), while Hispanic respondents were more willing than White respondents (80% vs. 75%, *p* < 0.003). Females were less likely than **makes** (72% vs. 79%, *p* < 0.001).

**Correct**

- Most respondents were willing to get the vaccine for themselves (75%) or their children (73%). Notably, Black respondents were less willing than White respondents (47% vs. 79%, *p* < 0.001), while Hispanic respondents were more willing than White respondents (80% vs. 75%, *p* < 0.003). Females were less likely than **males** (72% vs. 79%, *p* < 0.001).
